# Age and Interleukin-15 Levels Are Independently Associated With Intima-Media Thickness in Obesity-Related NAFLD Patients

**DOI:** 10.3389/fmed.2021.634962

**Published:** 2021-05-21

**Authors:** Giovanni Tarantino, Vincenzo Citro, Clara Balsano, Domenico Capone

**Affiliations:** ^1^Department of Clinical Medicine and Surgery, Federico II University Medical School of Naples, Naples, Italy; ^2^Department of General Medicine, “Umberto I” Hospital, Nocera Inferiore, Italy; ^3^Department of Clinical Medicine, Life, Health and Environmental Sciences-MESVA, University of L'Aquila, L'Aquila, Italy; ^4^Clinical Pharmacology Consultant, Casoria, Italy

**Keywords:** age, interleukin 15, intima-media thickness, non-alcoholic fatty liver disease, monocyte chemoattractant protein-1, granulocyte-macrophage colony-stimulating factor, basic fibroblast growth factor, C reactive protein

## Abstract

Common carotid intima-media thickness (IMT) represents a functional and structural marker of early, precocious, and subclinical atherosclerosis, independently from the carotid plaque. Macrophage cells, which have been detected in adipose tissue and atherosclerotic plaques, are regulated by interleukin-15 (IL-15). At the light of the conflicting results concerning the role of IL-15 in atherosclerosis, the aim of the study was to retrospectively evaluate in a population of 80 obese patients, with median age of 46 years (IQR 34–53 years), with a low rate of comorbidities but with non-alcoholic fatty liver disease (NAFLD) or hepatic steatosis (HS), the relationship between IMT and serum concentrations of IL-15. Anthropometric measures, metabolic profile, and serum inflammatory markers, as well as the levels of IL-15, MCP-1, b FGF, and GM-CSF, were analyzed by a bead-based assay. IMT, HS, subcutaneous, and visceral adipose tissues were detected by ultrasonography. The IL-15 levels of the obese patients were increased with respect to those of 44 young healthy subjects, i.e., 2.77 (1.21–4.8) vs. 1.55 (1–2.4) pg/mL (*P* = 0.002). In the univariate analysis, IL-15 levels were associated to IMT and to those of MCP-1, b FGF, and GM-CSF, without any relation to other inflammatory markers such as CRP and ferritin, except fibrinogen. In the multivariate analysis, after adjusting the HS severity for the extent of visceral adiposity, a dramatic change in prediction of IMT by HS was shown (β from 0.29 to 0.10, *P* from 0.008 to 0.37). When the visceral adipose tissue was combined with IL-15, on the one hand, and the well-known coronary artery disease (CAD) risk factors—i.e., age, gender, smoking status, HDL-cholesterol concentrations, triglycerides levels, and HOMA—on the other, only age and IL-15 remained the predictors of IMT (β = 0.60, *P* = 0.0001 and β = 0.25, *P* = 0.024, respectively). There was no association of IL-15 with various anthropometric parameters nor with body fat distribution and severity of HS, also after adjusting for age. Age is resulted to be the main factor in the prediction of IMT and thus of early atherosclerosis. The prediction of IMT by IL-15 coupled with the lack of prediction by the well-known CAD risks is in agreement with recent data, which emphasizes the main role of the immune system in the onset/worsening of atherosclerosis, even though the role of visceral adiposity should be further deepened. Age and IL-15 levels were both predictors of early atherosclerosis in this population of obese patients with NAFLD, suggesting a possible role of this cytokine in the atherosclerosis process.

## Introduction

Aging is an independent risk factor for the development of atherosclerosis (1). Common carotid intima-media thickness (IMT) is recently recognized as a non-invasive diagnostic tool for the identification of precocious (early) or subclinical atherosclerosis (2). This process is initiated by oxidized low-density lipoprotein (OxLDL) causing inflammation and signaling monocytes to enter the arterial wall after transforming themselves into macrophages. As a regulator of those cells (3), interleukin-15 (IL-15) is overexpressed in atherosclerotic lesions in both humans and ApolipoproteinE (ApoE)-deficient mice (4). Specifically, IL-15 mRNA was found in the fibro-lipid and lipid-rich plaques associated with OxLDL-positive macrophages (5). Contextually, serum IL-15 concentrations are significantly higher in patients with coronary artery disease (CAD) than in healthy subjects (6). Moreover, IL-15 genetic variants have been linked to an increased risk of CAD (7) and metabolic syndrome (MS) (8). Recent data are in favor of circulating levels of IL-15 reflecting the visceral distribution of adipose tissue (9). IL-15 regulates fractalkine-CX3CR1 chemokine signaling, which is involved in atherogenesis, promoting aortic smooth muscle cell proliferation and intimal thickening (10, 11).

In contrast to previous observations showing a pro-atherogenic effect of IL-15, the authors found that the blockade of endogenous IL-15 increased intima thickening (10). What is more, some experiments *in vitro* suggest that IL-15 may contribute to atherosclerotic plaque integrity, avoiding its rupture (12). Coming back to the inner role of monocytes/macrophage interaction in obesity and, consequently, atherosclerosis, the evidence in favor of a dominant role in chronic inflammation exerted by the monocyte chemoattractant protein-1 (MCP-1) has been repeatedly provided (13). Similarly, the granulocyte-macrophage colony-stimulating factor (GM-CSF) participates in a non-resolving inflammatory response that leads to subendothelial expansion (14). When dealing with basement membranes/subendothelial extracellular matrix of blood vessels, the basic fibroblast growth factor (b FGF), secreted by human adipocytes, bears a noticeable importance as an angiogenetic factor (15, 16), predicting future CAD occurrence (17). On the basis of the evidence that obese patients with ectopic fat storage, i.e., non-alcoholic fatty liver disease (NAFLD) or hepatic steatosis (HS), are at an increased CAD risk, and at the light of the conflicting results (6, 7, 10, 12) concerning the role of IL-15 in atherosclerosis, we aimed at evaluating in obesity-related NAFLD patients the serum concentrations of this cytokine and its association with IMT. Furthermore, we searched for each of the relationships between levels of IL-15 and (i) the markers of inflammation, (ii) the monocyte/macrophage-linked cytokine/chemokines, such as MCP-1 and GM-CSF, and (iii) the angiogenetic factor b FGF. To complete the analysis, any possible link was sought between IL-15 concentrations and body fat distribution, insulin resistance (IR), and lipid profile.

## Methods

In this observational (retrospective) substudy, we used the same patient sample contained in a previous research (18) according to The International Committee of Medical Journal Editors (IC-MJE) at http://www.icmje.org/recommendations/browse/publishing-and-editorial-issues/overlapping-publications.html.

### Population

For the current analysis, we used data drawn from records of 80 obese patients with NAFLD, who formed the population of the study. This sample had been on a balanced low-calorie, low-fat (25% of calories) diet for 3 months prior to study and was characterized by sedentary lifestyle. Forty-four young healthy subjects had been previously evaluated to set the range of IL-15.

### Body Fat Distribution and Liver Fat Storage at Ultrasound

The degrees of obesity (I–II–III, i.e., light, moderate, and severe) were established on the basis of BMI cutoff points of 30–34.9, 35–39.9, and >40 kg/m^2^, respectively. Visceral obesity was identified by measuring waist circumference (WC) at the midpoint between the lower border of the rib cage and the iliac crest. Hip circumference was measured around the widest part of the buttocks, with the tape parallel to the floor, and the waist-to-hip ratio (WHR) was calculated.

Subcutaneous adipose tissue (SAT) was defined as the thickness between the skin–fat interface and the linea alba, avoiding compression. Visceral adipose tissue (VAT) was defined as the distance between the anterior wall of the aorta and the internal face of the recto-abdominal muscle perpendicular to the aorta, measured 1 cm above the umbilicus (19).

The classification of “bright liver” or HS was based on the following scale of hyperechogenity: grade 0 = absent, grade 1 = light, grade 2 = moderate, and grade 3 = severe, pointing out the difference between the densities of the liver and the right kidney (20, 21).

### IMT Evaluation

The common carotid artery, the carotid bulb, and the near and far wall segments of the internal carotid artery were bilaterally scanned by ultrasound. Images were obtained in longitudinal sections with a single lateral angle of insonation, optimizing the image for the far wall. IMT was defined as the distance between the interfaces of the lumen-intima and media-adventitia. Six manual measurements were performed, with automatic border detection, at equal distances along 1 cm on the far wall of the common carotid, according to the consensus statement from the American Society of Echocardiography Carotid Intima-Media Thickness Task Force, endorsed by the Society for Vascular Medicine (22).

### Coronary Artery Disease Risk Factors

Systolic/diastolic blood pressure (SBP/DBP) was the average of three consecutive detections taken after having allowed the subjects to rest for 5 min in the sitting position.

Smoking status was assessed by an interview, and patients were categorized as active, passive, past, and no smokers.

### Criteria for Diagnosis of NAFLD

Obese patients, independently of evident hepatic cytolysis, were diagnosed as having NAFLD if they satisfied (i) an inclusion criterion, i.e., the presence of hyperechogenity, the so-called “bright liver,” based on a three-grade scale at US, as discussed below; and (ii) an exclusion criterion, i.e., the absence of any viral, autoimmune, metabolic disease, e.g., Wilson disease, hemochromatosis, or anti-trypsin deficiency, which were ruled out by appropriate testing, following the generally accepted diagnostic guidelines. Furthermore, in case of hypertransaminasemia, celiac disease was excluded estimating IgA anti-tissue transglutaminase antibodies. Alcohol abuse was screened according to DSM-IV diagnostic criteria and by means of the MAST (Michigan Alcohol Screening Test) and CAGE (Cut down, Annoyed, Guilty, Eye opener) tests, as well as by random tests for blood alcohol concentrations and the use of surrogate markers such as mean corpuscular volume.

### Inflammatory Markers

C reactive protein (CRP) values were determined by a high-sensitivity ELISA test, with reference values between 0.3 and 8.6 mg/L in healthy men and between 0.2 and 9.1 mg/L in healthy women (BioCheck, Inc., CA, USA). Ferritin and fibrinogen were performed by in-house standard procedures.

### Metabolic Profile

Serum triglycerides (TG), high-density lipoprotein cholesterol (HDL-cholesterol), and basal insulin were determined by in-house standard procedures (18). Low-density lipoprotein cholesterol (LDL-cholesterol) was calculated by the Friedewald formula as follows: total cholesterol – [HDL-cholesterol + (triglycerides/5)]. The IR status was determined by the homeostatic metabolic assessment (HOMA), which was assessed by the following formula: fasting insulin (μU/mL) × fasting glucose (mg/dL)/405 (23).

### Liver Enzymes

Alanine aminotransferase (ALT), pseudo cholinesterase (PCH), alkaline phosphatase (AP), and Gamma glutamyl transpeptidase (Gamma-GT) were analyzed by in-house standard procedures (18).

### Bead-Based Assay

Human IL-15 singleplex was performed according to the Bio-Rad systems protocol (Bio-Rad Lab., Inc., Hercules, CA, USA) as elsewhere reported (24, 25).

The coefficient of variation, calculated by standard deviation (SD)/mean × 100, for the intra-assay and inter-assay was <10% and <12%, respectively.

The control means were 0.14 ± 0.13 pg/mL for GM-CSF, 7.04 ± 2.01 pg/mL for b FGF, and 16.24 ± 15.73 pg/mL for MCP-1, respectively.

### Statistics

When analyzed by the Shapiro–Wilk test, variables not normally distributed were expressed as median plus 25–75 interquartile range (IQR) or 95% confidence intervals (CI) of the median; those derived from a normally distributed population were reported as mean plus SD. The difference between medians of two groups was detected by the Mann–Whitney test. When comparing throughout the three grades of HS or the three degrees of obesity, the ANOVA Kruskal–Wallis test with *post-hoc* analysis, the Conover–Inman test, was evaluated. When the ANOVA analysis was adjusted for age, the ANCOVA test was applied. In the univariate analysis performed as an exploratory step, the simple linear regression analysis (least squares) was used evaluating the coefficient with its standard error, 95% CI, and the *t* (*t*-value). To establish the best association of independent variables predicting the dependent variable, multiple regression was adopted (backward stepwise selection). Furthermore, to get the sense of which variables contribute more or less to the regression equation, the magnitude of the standardized coefficient beta (β) was calculated. *R*-square as a statistical measure of how close the data are to the fitted regression line was studied. To better address the problem of collinearity, three methods were carried out: the evaluation of the variance inflation fact (VIF), the tolerance (*T*), and the condition number (also labeled cond. index), setting the relative acceptable value, i.e., absence of collinearity, at (VIF <10, *T* > 0.1, and cond. index <10).

Factor analysis was applied to detect the structure in the relationships between variables, selecting a subset of them having the highest correlations with the principal component factors. The critical value was calculated by doubling Pearson's correlation coefficient for a 1% level of significance (5.152)/square root of the total population minus 2, i.e., (78) = 0.583. The power analysis to establish the minimum sample size concerning the relation of IL-15 to IMT was made adopting the value of Rho at Spearman's test with a type I error (alpha, significance) at 0.10 and type II error (beta, 1-power) at 0.20, resulting in 71 patients. ROC analysis (DeLong method) was used as a diagnostic decision-making. Indicatively, to measure the performance of the binary classification test (index test), the best cutoff was studied, coupled with the sensitivity, specificity, positive likelihood ratio (LR+) = sens/(1-spec), and the negative likelihood ratio (LR–) = (1-sens)/spec), pointing out that the more the LR+ is >1, the more likely the outcome. On the contrary, the more that the likelihood ratio for a negative test is <1, the less likely the outcome becomes. Furthermore, the correct classification percentage of IL-15 as well as other parameters and the area under the receiver operating characteristic (AUROC/AUC) were performed to evaluate the most appropriate models (the highest specificity and sensitivity) under the non-parametric assumption. The best cutoff (cut-point) of IL-15 with the highest specificity and sensitivity was calculated by means of the Youden index. The test equality of more ROC areas was performed to compare the performance of several variables.

A *P*-value <0.05 was accepted as the limit of significance.

Statistics was run on Stata 16.1 (Stata Corp., 4905 Lakeway Drive, College Station, Texas 77845, USA).

## Results

### Prevalence

The mean age ± SD of healthy subjects (20 males and 24 females) was 21 ± 3 years. The median age plus IQR of the obese was 46 (34–53) years. Patients of the studied population mainly suffered from moderate/severe obesity (*n* 62, 77.5%) and mild/moderate grade of HS (*n* 72, 90%, Table 1).

**Table 1:** Characteristics of the obese patients with NAFLD (*n* 80), comprehending the metabolic parameters.

Gender (M/F)	36/44	HDL-cholesterol males (mg/dL)	42.7 ± 9.0
Obesity degree I/II/III (n)	8/26/46	TG (mg/dL)	123.5 (83.5–188.0)
BMI	42.3 (38.1–46.8)	CRP (mg/mL)	0.56 (0.27–1.3)
WC females (cm)	118.9 ± 12.5	Fibrinogen (g/L)	295.5 (256.0–357.5)
WC males (cm)	118.9 ± 12.5	Ferritin females (ng/mL)	41.5 (20.0–69.0)
WHR females	0.95 (0.93–0.97)	Ferritin males (ng/mL)	167.5 (85.0–234.0)
WHR males	0.98 (0.96–1.0)	LDL-cholesterol (mg/dL)	66.8 (33.3–140.0)
IMT ≥ 0.09 cm (*n*)	48	IMT (cm)	0.09 (0.07–0.11)
SAT (cm)	2.6 (2.1–3.1)	GM-CSF (pg/mL)	2.01 (0.14–10.75)
VAT (cm)	7.5 (6.0–9.4)	B FGF (pg/mL)	58.68 (12.5–96.29)
ALT (U/L)	28 (21.5–29.0)	MCP-1 (pg/mL)	18.01 (0.14–56.68)
CHE (U/L)	9,671.4 ± 1,882.2	Fasting insulin (μU/mL)	10.9 (7.5–15.8)
AP (U/L)	73.0 (61.0–91.0)	Fasting glucose (mg/dL)	96.5 (87.0–114.0)
γ-GT (U/L)	25.0 (16.5–42.5)	HOMA	2.78 (1.85–4.18)
HS grade at US 1/2/3 (*n*)	22/50/8	HDL-cholesterol females (mg/dL)	46.4 ± 11.8
SBP (mm Hg)	130 (120–140)	DBP (mm Hg)	80 (80–90)
Active smokers (*n*)	32	Passive smokers	10

*WC, waist circumference; BMI, body mass index; WHR, waist-to-hip ratio; SAT, subcutaneous adipose tissue; VAT, visceral adipose tissue; HDL-cholesterol, high-density lipoprotein cholesterol; TG, triglycerides; CRP, C reactive protein; US, ultrasound; ALT, alanine aminotransferase; CHE, cholinesterase; AP, alkaline phosphatase; Gamma-GT, Gamma-glutamyl transglutaminase; LDL-cholesterol, low-density lipoprotein cholesterol; HOMA, homeostatic metabolic assessment; SPP, systolic blood pressure; DBP, diastolic blood pressure; IMT, intima-media thickness; GM-CSF, granulocyte-macrophage colony-stimulating factor; b FGF, basic fibroblast growth factor; MCP-1, monocyte chemoattractant protein-1; n, number*.

The values of liver enzymes in this group of obese patients with US features of liver fat storage were normal or slightly elevated. Moreover, the absence of hypertension, the metabolic profile not particularly altered, and the borderline median IMT showed that the studied cohort was homogeneously selected without advanced atherosclerosis.

When evaluating a well-known CAD risk factor, i.e., smoking status among 80 patients selected in this study, 32 subjects (40%) were classified as active smokers and 10 (12.5%) as passive smokers. The remaining obese patients were non-smokers (*n* 22) or past smokers (*n* 36). IMT was partially overlapping in the two groups, i.e., 0.09 (0.08–0.11) and 0.08 (0.08–0.10) median (95% CI of median), respectively, *P* = 0.60. There was a significant difference between IL-15 concentrations of obese patients and healthy subjects, i.e., median 2.77 (1.21–4.8) vs. 1.55 (1–2.4) pg/mL, *P* = 0.002. Assessing the serum concentrations of IL-15 in patients belonging to the three degrees of obesity and three grades of HS at US, no significant difference was found among the groups (*P* = 0.06 and *P* = 0.7, respectively) and also when adjusted for age (*P* = 0.06 and *P* = 0.6, respectively).

### Associations and Predictions (Statistical Model)

**Figure 1:**
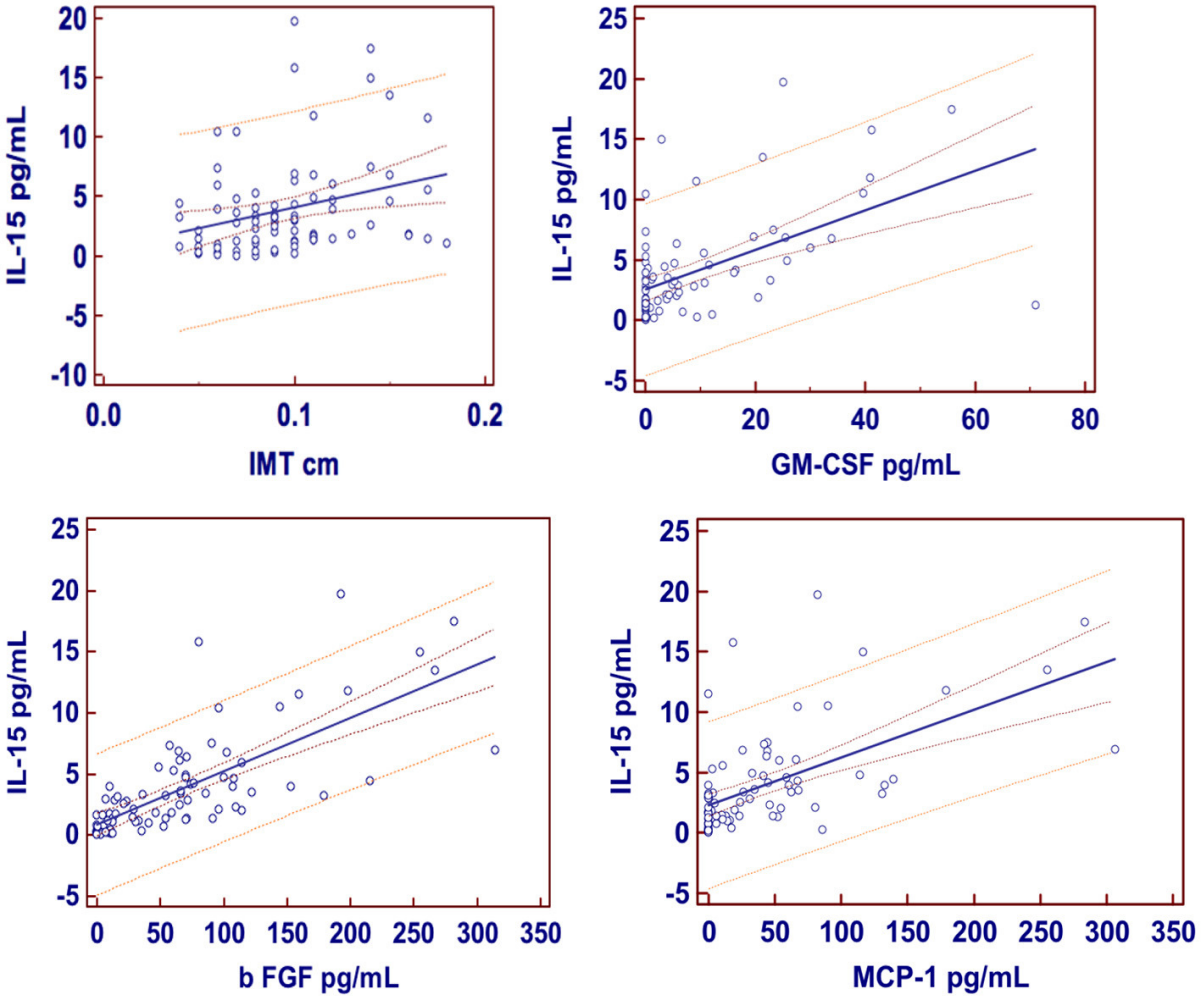
Regression equations. Just at the center the regression line is showed; 95% confidence interval as lines nearest to the regression line and 95% prediction interval the more distant ones. IMT, Intima-media thickness; GM-CSF, granulocyte-macrophage colony-stimulating factor; b FGF, basic fibroblast growth factor; MCP-1, monocyte chemoattractant protein-1.

Results of the univariate analysis (single linear regression): A significant association between the serum concentrations of IL-15 and IMT (Figure 1), also after adjusting for grades of HS (*t* = 2.65 vs. 2.59), was found. Furthermore, circulating concentrations of IL-15 were related to the levels of MCP-1, b FGF, and GM-CSF (Figure 1) without any relation to other inflammatory markers such as CRP and ferritin, with the exception of fibrinogen (Table 2). Serum concentrations of IL-15 were not related to BMI, WC, WHR, SAT, and VAT. IMT was predicted by the severity of HS at US (coefficient = 0.0017, standard error = 0.0006, CI = 0.004–0.029, *t* = 2.7050, *P* = 0.008).

**Table 2:** Predictions of IMT, CRP, fibrinogen, GM-CSF, b FGB, and MCP-1 by interleukin-15 levels.

	**Coefficient**	**Std. error**	**95% CI**	***t***	***P***	***R*^**2**^**
IL-15/IMT	35.51	13.41	8.8 to 62.22	2.65	0.009	0.08
IL-15/CRP	0.020	0.058	−0.096 to 0.136	0.34	0.73	0.01
IL-15/fibrinogen	0.013	0.006	0.0005 to 0.025	2.07	0.04	0.05
IL-15/GM-CSF	0.16	0.029	0.107 to 0.22	5.71	<0.001	0.29
IL-15/b FGF	0.044	0.0046	0.034 to 0.053	9.51	<0.001	0.53
IL-15/MCP-1	0.039	0.0062	0.027 to 0.052	6.32	<0.001	0.34

*Single linear regression using interleukin-15, IL-15, as the dependent variable. IMT, Intima-media thickness; CRP, C reactive protein; GM-CSF, granulocyte-macrophage colony-stimulating factor; b FGF, basic fibroblast growth factor; MCP-1, monocyte chemoattractant protein-1; Std. error, standard error; R^2^, R-square. The low R-square, in the presence of significance, shows that even noisy, high variability data (data points fall further from the regression line in the graph) can have a significant trend*.

The strength of the prediction of IL-15 on IMT, when adjusted for age, was moderately modified, with a difference of 15%, remaining significant vs. age (coefficient = 0.0016, standard error = 0.0007, β = 0.25, *t* = 2.3, *P* = 0.024). Age was the most accurate predictor of early atherosclerosis (coefficient = 0.0014, standard error = 0.0002, β = 0.60, *t* = 6.56, *P* = 0.0001).

As a collateral finding, in the univariate analysis, WC was clearly associated with VAT at US (coefficient 0.097; standard error 0.021; 95% CI 0.46–4.57; *P* ≤ 0.001).

Results of the multivariate analysis: When assessing the importance of some anthropometric/US parameters of visceral adiposity impacting on IMT, i.e., WC, WHR, and VAT, only VAT showed predictability for IMT (coefficient = 0.0069, standard error = 0.0019, β = 0.38, *t* = 3.54, *P* = 0.0007). Adding VAT to IL-15 and other well-recognized CAD risk factors, i.e., age, gender, smoking status, HDL-cholesterol concentrations, and triglycerides levels, only age and IL-15 remained the predictors for IMT. Insulin resistance, evaluated as HOMA, also included in the model, scarcely contributed to the regression equation (Table 3).

**Table 3:** Prediction of IMT by IL-15 and other CAD risk factors.

**Independent variables**	**Coefficient**	**Std. error**	**β**	***T***	***P***	**VIF**	***T***
Age	0.0015	0.0002	0.60	6.7	<0.0001	1.02	0.97
HOMA	0.0014	0.00075	0.21	1.89	0.06	1.01	0.98
IL-15	0.0015	0.0007	0.24	2.12	0.037	1.03	0.96

*Multiple regression equation (backward stepwise selection) using intima-media thickness (IMT) as the dependent variable. IL-15, Interleukin-15; HOMA, homeostatic metabolic assessment; Std. error, standard error; VIF, variance inflation factor; T, tolerance. The condition number was 8.57. The R-square of this regression was 0.44, indicating that the model well fits our data*.

Among CCL2/MCP-1, BFGF, GM-CSF, and IL-15, only the last one well predicted IMT.

Results of factor analysis: Values reported in Table 4 showed that HS severity at US was related to the adiposity degree, independently of the distribution, i.e., WC, BMI, SAT, and VAT (factor 1), but surprisingly showed no correlation with the common carotid IMT. Vice versa, IMT was related to age, as clearly showed in factor 2. The lack of the relation concerning liver fat excess to IMT was confirmed at the light of the dramatic change in the prediction of IMT by HS after adjustment for VAT (Table 5).

**Table 4:** Hidden relationships detected by the factor analysis.

	**Factor**	**1**	**2**
1	WC	**0.81**	0.17
2	BMI	**0.77**	0.009
3	HDL	−0.02	−0.44
4	TG	0.09	0.45
5	CRP	0.47	−0.09
6	Fibrinogen	0.33	−0.32
7	HOMA	0.32	0.18
8	SAT	**0.59**	−0.45
9	HS at US	**0.67**	0.45
10	VAT	**0.69**	0.51
11	IMT	**0.075**	**0.77**
12	Age	0.039	**0.67**
**Percent of total variance explained by factors**			
**Factor**	**1**	**2**	
%	24.7	19.1	

*Factor analysis; rotated loading matrix (VARIMAX, Gamma = 1.0). The critical value was calculated by doubling Pearson's correlation coefficient for a 1% level of significance (5.152)/square root of the total population minus 2, i.e., (78) = 0.583. WC, Waist circumference; BMI, body mass index; HDL, high-density lipoprotein cholesterol; SAT, subcutaneous adipose tissue; VAT, visceral adipose tissue; CRP, C reactive protein; US, ultrasound; HOMA, homeostatic metabolic assessment; TG, triglycerides; IMT, intima-media thickness; HS, hepatic steatosis; US, ultrasound. It should be stressed that both the factors explained nearly half the total variance. The hidden relationships were evidenced in bold*.

**Table 5:** Prediction of IMT by HS of the univariate analysis.

**Independent variables**	**Coefficient**	**Std. error**	**β**	***t***	***P***		
HS	0.017	0.0062	0.29	2.7	0.0084		
Prediction of IMT by HS after adjustment for VAT of the multivariate analysis.							
**Independent variables**	**Coefficient**	**Std. error**	**β**	***t***	***P***	**VIF**	***T***
HS	0.007	0.008	0.10	0.89	0.37	1.76	0.56
VAT	0.003	0.002	0.20	1.78	0.079	1.76	0.56

*IMT, Intima-media thickness; VAT, visceral adipose tissue; HS at ultrasound, hepatic steatosis; Std. error, standard error; VIF, variance inflation factor; T, tolerance. The condition number was 9.48. The R-square of the regression of the univariate analysis was 0.086 and that of the multivariate analysis was 0.12 indicating that the models moderately fit our data*.

### Concordance

The intra-/inter-observational variability of US estimates did not reach significance, with the mean difference being equal to 1.7, 2.2, 2.5, and 1.9%, and 2.1, 3.3, 3.9, and 3.1% for the HS, VAT, SAT, and common carotid IMT, respectively, with an ρc of 0.92.

### Sensitivity Analysis

**Figure 2:**
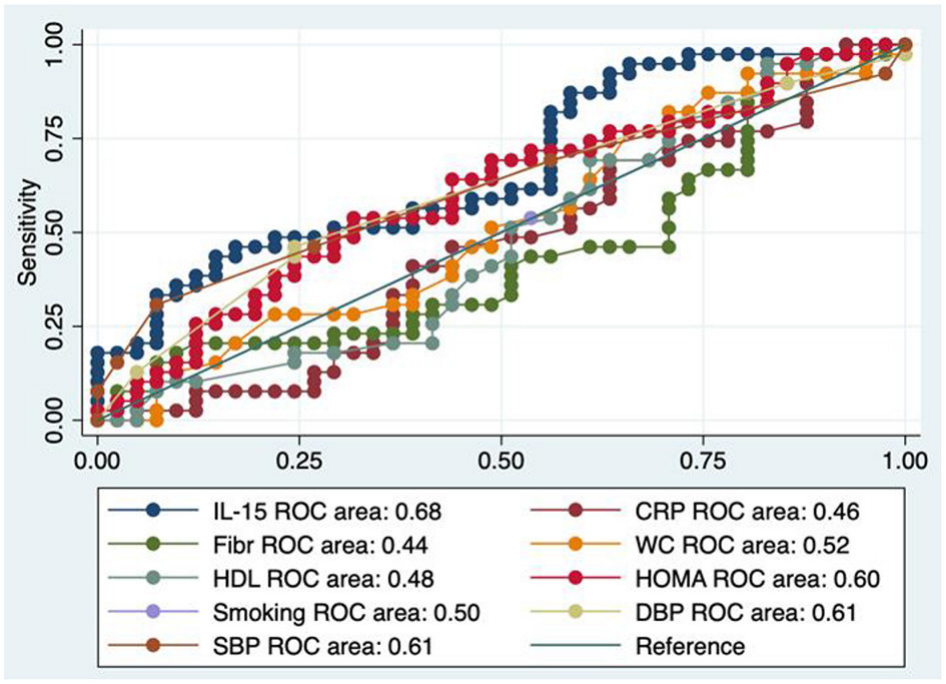
Test equality of more ROC areas. IL-15, interleukin-15; Fibr, fibrinogen; CRP, C reactive protein; WC, waist circumference; SBP, systolic blood pressure; DBP, diastolic blood pressure; HOMA, homeostatic metabolic assessment; HDL, HDL cholesterol.

Looking at Figure 2, showing the basal model of the ROC area confronted with those of other parameters, concerning the diagnostic accuracy of IL-15 toward early atherosclerosis, we evidenced that IL-15 AUC performed in a modest way, although a little better than that of the classical CV risk factors. The cutoff of IL-15 resulted to be 2.53 pg/mL with a sensitivity of 59%, a specificity of 54%, a correct classification of 56%, a positive likelihood of 1.27, and a negative likelihood of 0.76; area under ROC curve = 0.68.

## Discussion

An interesting result of this retrospective study is that the serum concentrations of IL-15 were increased and clearly linked to IMT. Carotid IMT, arterial stiffness (26), coronary artery calcification (27), endothelial dysfunction (28), and impaired left ventricular function (29) represent an intermediate phenotype of early atherosclerosis and are predictors of cardiovascular events (30). We used B-mode ultrasonography for detection of early (IMT) but not late (plaque morphology) atherosclerotic disease (31). Comparing the results of our study with recent findings, we were unable to confirm an independent role of GM-CSF, b FGF, and MCP-1 in predicting early atherosclerosis, evaluated as IMT. Nevertheless, the association between IL-15 and GM-CSF, b FGF, and MCP-1, showed by our results, allows us to hypothesize that these molecules could play a role in a delayed phase of atherosclerosis. In fact, the results suggested important roles for G-CSF in the mobilization of bone marrow stem cells altering plaque development (32). Similarly, the collateral development induced by b FGF exceeded the VEGF-induced one in the process of arteriogenesis (33). Finally, MCP-1 could further impact on fully developed atherosclerosis via hypertension (34). In the era of personalized medicine, it is of utmost importance to be able to identify subjects at the highest cardiovascular (CV) risk. To date, single biomarkers have failed to markedly improve the estimation of CV risk. Using novel technology, simultaneous assessment of large numbers of biomarkers may hold promise to improve prediction (35). We would like to emphasize that only the subclinical, early atherosclerosis and not its severity was evaluated in our study based on obese patients suffering from NAFLD by appreciating the common carotid IMT (36).

We found no relation of IL-15 to various anthropometric parameters, such as BMI, WC, WHR, as well as US features of body fat distribution (with the exception of VAT), and to severity of HS, also after adjusting for age. This lack of correlation strengthens the independent role of IL-15 on IMT, avoiding the interference of a well-known CAD risk, that is, obesity. It is noteworthy to stress that hypercholesterolemia leads to the upregulation of IL-15 within spleen, and blood DNA vaccination against IL-15 markedly reduces atherosclerotic lesion size but does not promote lesion stability (37).

Discussing possible mechanisms and explanations for the link between circulating levels of IL-15 and IMT, we hypothesize that IL-15 acts as a potent mediator in immune reactions involved in early atherosclerosis by increasing cytokines/chemokines production both from fibroblasts and macrophages of vessel origin. Nevertheless, the adipose tissue, for its characteristics of dynamic organ with endocrine–autocrine and paracrine action, contributes to the cytokines/chemokines network. In fact, the impact of visceral fat on IMT was somehow present in our series, even though was somehow obscured by age.

Due to the lack of data in obese patients without NAFLD to be confronted with our severely obese patient population (median BMI 42.3), mainly because there was some difficulty enrolling them, we were not able to do this comparison. Despite the controversial evidence that NAFLD *per se* increases the cardiometabolic risk (38–40), it is worth underlining that, in our population, the excess fat deposition in liver was linked to increased IMT only when its severity was not adjusted for abdominal adiposity (VAT). In other words, the presence of abdominal adiposity attenuates the role of HS on IMT. This finding could be explained by hypothesizing that mechanisms, beyond low-grade chronic inflammation, i.e., hormones, growth factors, and adipokines imbalance—contributing to the liver fat excess—play a major role on the atherosclerotic process. Although other authors have recently demonstrated a role for MCP-1 in obesity-induced IR (41, 42), a cardiometabolic factor associated with global CAD risk, we failed to find a link between MCP-1 and IMT.

In light of normal or slightly elevated values of liver enzymes of the obese with US features of liver fat storage, the absence of hypertension, the metabolic profile not particularly altered, and the borderline median IMT, we can hypothesize that, given these characteristics, our patients belonged to a specific cohort without advanced atherosclerosis. Furthermore, patients were middle-aged. In other words, our population comprehended both healthy and unhealthy obese patients, being hepatic steatosis severity the divide between them (43).

It is noteworthy that the association between age and IMT in our cohort was in agreement with ongoing research, demonstrating that gender and age were stronger predictors of IMT (44). We recognize that the statistical relevance of prediction of IMT by IL-15 is partially decreased when adjusting for demographic data. The evidence of prediction of IMT by IL-15 without age could lead to an overestimation of its role of IL-15. The partial overlap of the two predictions is justified by the importance of age in the onset/progression of the atherosclerotic process. Finally, recent research highlights the main role of IL-15 in promoting inflammation in adipose tissues, which, in turn, determines chronic low-grade inflammation that is at the basis of obesity-associated metabolic syndrome (45).

It is not of limited importance the impact of IL-1b in atherosclerosis, but likely this cytokine plays a major role in advanced clinical stages of atherosclerosis, such as angina, myocardial infarction, and cerebral stoke, as evident in literature (46–51). On the contrary, our data were derived by exploring the serum concentrations of IL-15 in patients who did not present with established coronary heart disease.

Finally, we stress that the sensitivity analysis concerning the role of IL-15 in correctly diagnosing the presence of early atherosclerosis resulted in a moderate reliability.

## Limitations

We are not able to exclude a selection bias in our study due to the fact that we analyzed a previous study characterized by a rigorous selection of the obese patients. The severity of carotid atherosclerosis was not evaluated in the present study, but it will be taken into serious account in a future study (52, 53). In the univariate analysis, the prediction of IMT by IL-15 resulted to be highly significant. But in a multivariate model comprehending other CAD risk factors such as VAT, age, gender, smoking status, HDL-cholesterol concentrations, triglycerides levels, and HOMA, the strength of this prediction somehow decreased. Despite a balanced diet being common to all patients, this could have modified the metabolism and, consequently, the inflammatory homeostasis of our patients. It is noteworthy to stress that maintaining the obese to a free diet would be ethically unacceptable. Another limitation was the lack of adiposity evaluated by MRI. Finally, having obese patients who refused to undergo liver biopsy, the diagnosis of HS by US could have been biased, although recent studies emphasize the reliability of this tool (54).

## Conclusion

This retrospective study shows that, in addition to age (prominent factor), levels of IL-15 are associated with IMT in obese patients with NAFLD suggesting a possible role of this cytokine in the atherosclerosis process, although its diagnostic performance is discrete.

## Future Directions

CAD, a leading risk for mortality all over the world, recognizes atherosclerosis as the main underlying cause. Being atherosclerosis reckoned as a chronic inflammatory disease of blood vessels and considering that lesions of atherosclerosis contain macrophages, T cells, and other cells of the immune response, together with cholesterol that infiltrates from the blood, studies are now under way to develop new therapies based on these concepts of the involvement of the immune system (mainly of pro-inflammatory cytokines from macrophage) in atherosclerosis.

Specifically, our results could lend credence to further therapeutical approach, i.e., human immunoglobulin monoclonal antibody directed against the human pro-inflammatory cytokine IL-15 that is associated with a variety of autoimmune and inflammatory disorders, as for some aspects, atherosclerosis could be rethought (55).

However, there is still much to learn about immune cells and their mechanisms affecting atherosclerosis.

## Data Availability Statement

The original contributions generated for the study are included in the article/supplementary material, further inquiries can be directed to the corresponding author/s.

## Ethics Statement

Ethical review and approval was not required for the study on human participants in accordance with the local legislation and institutional requirements. Written informed consent for participation was not required for this study in accordance with the national legislation and the institutional requirements. The paper does not report on primary research. Our analysis looked at data of this cohort, respecting complete anonymity and was performed internally as part of an evaluation to improve our quality of care. Patients were diagnosed and treated according to national guidelines and agreements. Testing blood as well as recording all other variables included in our analysis was essential for confirming diagnosis and classifying patients. It was done for each patient without fail and as part of routine care, and was in no way an add-on for purposes of research. For these reasons, no ethical approval was requested, and informed written consent was not obtained from each subject.

## Author Contributions

GT: conceptualization, data analysis, statistics, and writing manuscript. VC: data analysis contribution. CB: valid criticism providing. DC: design study contribution. All the authors discussed the data and approved the final version of the manuscript.

## Conflict of Interest

The authors declare that the research was conducted in the absence of any commercial or financial relationships that could be construed as a potential conflict of interest.

## Abbreviations

IMT: intima-media thickness
OxLDL: oxidized low-density lipoprotein
IL-15: interleukin-15
ApoE: ApolipoproteinE
CAD: coronary artery disease
MS: metabolic syndrome
MCP-1: monocyte chemoattractant protein-1
GM-CSF: granulocyte-macrophage colony-stimulating factor
b FGF: basic fibroblast growth factor
NAFLD: non-alcoholic fatty liver disease
HS: hepatic steatosis
IC-MJE: International Committee of Medical Journal Editors
WC: waist circumference
WHR: waist-to-hip ratio
SAT: subcutaneous adipose tissue
VAT: visceral adipose tissue
MAST: Michigan Alcohol Screening Test
CAGE, Cut down, Annoyed, Guilty: Eye opener
CRP: C reactive protein
TG: triglycerides
HDL-cholesterol: high-density lipoprotein cholesterol
LDL-cholesterol: low-density lipoprotein cholesterol
HOMA: homeostatic metabolic assessment
ALT: alanine aminotransferase
PCH: pseudo cholinesterase
AP: alkaline phosphatase
Gamma-GT: Gamma glutamyl transpeptidase
US: ultrasound
IQR: interquartile range
CI: confidence interval
SD: standard deviation
*t*: *t*-value.
